# Construction of a Fibroblast-Associated Tumor Spheroid Model Based on a Collagen Drop Array Chip

**DOI:** 10.3390/bios11120506

**Published:** 2021-12-09

**Authors:** Hyewon Roh, Hwisoo Kim, Je-Kyun Park

**Affiliations:** 1 Department of Bio and Brain Engineering, Korea Advanced Institute of Science and Technology (KAIST), 291 Daehak-ro, Yuseong-gu, Daejeon 34141, Korea; hyewonr@kaist.ac.kr (H.R.); hwiss@kaist.ac.kr (H.K.); 2 KAIST Institute for Health Science and Technology, 291 Daehak-ro, Yuseong-gu, Daejeon 34141, Korea

**Keywords:** 3D cell culture, droplet contact-based spheroid transfer, drug assay model, fibroblast, glioblastoma multiforme, spheroid invasion assay

## Abstract

Spheroid, a 3D aggregate of tumor cells in a spherical shape, has overcome the limitations of conventional 3D cell models to accurately mimic the in-vivo environment of a human body. The spheroids are cultured with other primary cells and embedded in collagen drops using hang drop plates and low-attachment well plates to construct a spheroid–hydrogel model that better mimics the cell–cell and cell–extracellular matrix (ECM) interactions. However, the conventional methods of culturing and embedding spheroids into ECM have several shortcomings. The procedure of transferring a single spheroid at a time by manual pipetting results in well-to-well variation and even loss or damage of the spheroid. Based on the previously introduced droplet contact-based spheroid transfer technique, we present a poly(dimethylsiloxane) and resin-based drop array chip and a pillar array chip with alignment stoppers, which enhances the alignment between the chips for uniform placement of spheroids. This method allows the facile and stable transfer of the spheroid array and even eliminates the need for a stereomicroscope while handling the cell models. The novel platform demonstrates a homogeneous and time-efficient construction and diverse analysis of an array of fibroblast-associated glioblastoma multiforme spheroids that are embedded in collagen.

## 1. Introduction

For basic and applied research of disease, efficient development of new drugs, innovative development of precision medicine, and reduction of animal experimentation, it is necessary to construct an in vitro cell model that precisely imitates the in vivo tumor environment. The conventional two-dimensional (2D) cell models significantly differ from the in vivo environment, producing cellular behavior dissimilar to that of natural conditions. This is because the cell-to-cell and cell-to-extracellular matrix (ECM) interactions within the actual body, as well as the expression of genes, are not properly achieved [1,2,3]. Thereby, three-dimensional (3D) cell culture models have recently been introduced and great advancements have been made in the field [4,5,6]. Out of the numerous types of 3D cell culture models, spheroid, which is a 3D aggregate of tumor cells in a spherical shape, is being actively explored due to its relatively simple construction and wide applicability in terms of cell types and uses [7,8]. Since spheroids can also be manipulated to mimic the 3D in vivo tumor structures, they have been used as a tumor model for various applications, such as investigating the mechanisms of drug response and delivery, testing anti-cancer effects, or even simulating a perfusable blood vessel network using microfluidic technologies [3,9,10,11]. In addition, many tumor spheroid-embedded hydrogel models have been built using collagen and matrigel to recapitulate the features and environment of the ECM that structurally and biochemically support the tumor cells [12,13,14,15,16,17]. By varying the composition, type, and organization of the protein-based hydrogel, the tumor spheroid-embedded hydrogel models serve as a useful platform for investigating invasive and metastatic characteristics, effects of drug treatment, and other biochemical cues that regulate tumor cell behavior [18].

Despite the wide applicability and use of the spheroid-embedded hydrogel models, the process of culturing spheroids and handling them for embedding into hydrogel has several limitations. Currently, the most common method of constructing a spheroid is the hanging drop method, which simply requires micro-sized wells where the liquid is deposited and hung as a drop. However, the operational procedures of this conventional technique are inefficient and complex because the relocation of the spheroid and the replacement of the medium are carried out using a pipette. Several studies have reported that the spheroids are collected using a pipette tip and embedded into the hydrogel for further analysis [3,12,19]. However, such a method of handling spheroids often results in well-to-well variation and spheroids being damaged or even lost [20,21,22].

Few studies were reported to overcome the labor-intensive and time-consuming problem of handling the spheroids by constructing agarose microwells with slopes of different angles to exchange the medium without damaging the spheroids [20]. Our group has also previously reported a novel droplet contact-based spheroid transfer (DCST) technique and array chips for simultaneous collection and transplantation of spheroids [23,24]. Despite research, the problem was not resolved as it was still difficult to remove the old cell medium using the slanted microwells while not contacting the spheroids with a pipette tip. In the case of the DCST, the array chips were appropriately made for exchanging spheroid medium without damaging the spheroid. However, the array chips still did not have any supporting frame, resulting in unstable alignment and an inconsistent shape of the spheroid-embedded hydrogel model. In addition, for a successful spheroid embedment into the hydrogel, the transfer procedure must be performed under magnifying equipment, which is tedious and time-consuming.

On this basis, this study presents an improved DCST method that simply and uniformly constructs an array of fibroblast-associated glioblastoma multiform spheroid models. Four sets of alignment stoppers have been developed and integrated into the original DCST method previously introduced [23,24] to culture and embed spheroids into hydrogel with perfect alignment. The performance of the improved DCST method with alignment stoppers is compared to that of the original DCST and conventional manual pipetting methods in the areas of spheroid shape, retention rate, and uniformity of the spheroid models. Then, the drop array chip (DAC) with alignment holes is used to co-culture both glioblastoma spheroids and fibroblast-associated glioblastoma spheroids using the chip-based hanging drop method. The spheroids are simultaneously relocated to new medium drops or into collagen drops using the pillar array chip (PAC) with alignment stoppers with stability and uniformity. Using the alignment stoppers, the alignment between the two chips is enhanced, eliminating the need to use magnifying equipment, such as a stereomicroscope, while the spheroids are being transferred. In addition, we have used the more invasive cell type and also associated fibroblast to investigate the invasive behavior of the tumor cells in our device, and since we have used a more invasive cell type with fibroblast, it is important that the alignment stoppers keep our spheroids in the center of the DAC well. Furthermore, the growth and invasion morphology of the glioblastoma spheroids are investigated under various conditions using the cell viability test or the collagen well chip (CWC). Finally, the effects of the ratio of co-cultured fibroblast cells and drug treatment are demonstrated and analyzed with the fibroblast-associated glioblastoma spheroid models.

## 2. Materials and Methods

### 2.1. Materials

For the fabrication of array chips for DCST, poly(dimethylsiloxane) (PDMS) monomers and curing agents were purchased from Dow Corning (Midland, MI, USA). Poly(methyl methacrylate) (PMMA, 3 mm thickness) plates were purchased from YM Tech (Daejeon, Korea). Phosphate-buffered saline (PBS), Dulbecco’s modified Eagle’s medium (DMEM), fetal bovine serum (FBS), penicillin/streptomycin (P/S) were purchased from Corning (Corning, NY, USA). Rat tail collagen type I was purchased from Corning. Calcein-AM and ethidium homodimer-1 (EthD-1) and Hoechst 33342 solution were purchased from Invitrogen (Carlsbad, CA, USA). CellTracker^TM^ Green CMFDA and CellTracker^TM^ Red CMTPX dyes for cell tracking were purchased from Thermo Fisher Scientific (Waltham, MA, USA). Doxorubicin hydrochloride for drug treatment was purchased from Sigma-Aldrich (St. Louis, MO, USA). Other chemicals and reagents were purchased from Sigma-Aldrich unless otherwise stated.

### 2.2. Device Fabrication

The molds of a DAC with alignment stopper holes and a PAC with alignment stoppers were all designed using the AutoCAD and Inventor program (Autodesk, San Rafael, CA, USA). Next, the objects were fabricated with clear photopolymer resins (plasCLEAR) using a 3D printer (The PICO2; Asiga, Alexandria, Australia). The PMMA plates for the 3D printed molds were fabricated by etching the PMMA plates into an appropriate pre-designed shape using a laser cutter (C40–60W; Coryart, Anyang, Korea). Then, to make a complete master mold, the 3D printed objects were attached to the etched PMMA plates with a double-sided adhesive tape (Kyodo Giken Chemical, Saitama, Japan).

To fabricate the PDMS-based array chips, including the DAC with alignment stopper holes and the PAC with alignment stoppers, the master molds were positioned using the alignment stoppers and held together in a place by placing two sets of magnets on each side of the master mold. Then, the master molds were covered using aluminum foil as a support structure. A PDMS mixture, which consists of a PDMS monomer and a curing agent at a ratio of 10:1, was poured onto the patterned mold. The molds were then degassed in a vacuum chamber for 30 min to remove all the air bubbles that could cause the surface of the final PDMS product to have an uneven surface. After placing the molds in a 65 °C dry oven and curing overnight, the DAC with alignment stopper holes and the PAC with alignment stoppers were peeled off gently from the supporting molds. Before use, the array chips were autoclaved for sterilization (Supporting Figure S1).

### 2.3. Two-Dimensional (2D) Cell Culture

For spheroid cell culture and analysis, the human glioblastoma multiforme cancer cell line U87-MG was purchased from the Korean Cell Line Bank (KCLB; Seoul, Korea). U87-MG cells were 2D cultured in a cell culture dish as a monolayer in DMEM supplemented with 10% FBS and 1% P/S. Then, the cells were maintained in a humidified incubator of 37 °C and 5% CO_2_ to be cultured. The medium was replaced every 48 h and the cells were subcultured when approximately 70% of the dish was full. The normal human lung fibroblast (NHLF) (Lonza; Basel, Switzerland) cell line was 2D cultured in a Fibroblast Growth Medium-2 (FGM-2), which consists of Fibroblast Basal Medium (FBM) and a reagent pack (HEPES, TNS, Trypsin/EDTA) (Lonza). The NHLF cell line was also 2D cultured in a humidified incubator of the same condition as U87-MG and the medium was replaced every 48 h.

### 2.4. Three-Dimensional (3D) Cell Culture and Embedment into Collagen

The two-dimensionally cultured U87-MG cells were suspended using a trypsinization process for spheroid formation, then were counted and diluted with an extra medium to meet the desired initial seeding concentrations (1000/2000/4000/8000 cells per well). Using a pipette, 24 μL of the cell suspensions of desired concentration were each loaded to the well of the DAC with alignment stopper holes. Then, the DAC with alignment stopper holes was positioned upside down on the two PMMA columns adhered to the culture dish as a hanging drop method. 7 mL of PBS was loaded at the bottom of the culture dish to prevent drop evaporation. The cell culture dish with the DAC with alignment stopper holes was placed in a humidified incubator of 37 °C and 5% CO_2_. The medium of the droplets, in which spheroids are constructed, is replaced every 48 h using the PAC with alignment stoppers.

Rat tail collagen type I was diluted to 3 mg/mL to 0.5 mg/mL using a NaOH solution, and 5 μL was loaded to each of the wells in a Collagen Well Chip (CWC) with alignment holes. The CWC was developed by modifying the height of the DAC and flattening the bottom of the well to enhance the observation of the invasive nature of the tumor spheroid under bright field conditions without using any chemical treatment (Supporting Figure S2). The PAC with alignment stoppers was used to transfer the spheroids in the DAC with alignment stopper holes to the collagen-filled well of the CWC. After contacting the PAC with CWC for 30 s to settle down the spheroids, the spheroid-embedded collagen was placed inside a cell culture dish and put inside the incubator for 30 min to polymerize the collagen drops. Subsequently, the entire cell culture dish with the CWC was loaded with 5 mL of cell culture medium, enough to immerse the entire chip. The medium was changed every 48 h.

### 2.5. Fibroblast-Associated Glioblastoma Spheroid Culture and Invasion Assay

The fibroblast-associated glioblastoma spheroids were similarly constructed as the glioblastoma spheroids. Both the two-dimensionally cultured U87-MG and NHLF cell lines were suspended by the trypsinization process, counted, and diluted with an appropriate medium to meet the seeding concentration ratios (e.g., U87-MG:NHLF = 2:1, 4:1, or 8:1). The cell suspensions of U87-MG and NHLF cells were gently mixed using a pipette accordingly, and 24 μL of the cell suspension mixture was seeded to each well of the DAC. Similar to the mono-cultured spheroids, the medium of the co-culture spheroids was also replaced every 48 h using the DAC with alignment stopper holes and the PAC with alignment stoppers. The spheroid-seeded DAC with alignment stopper holes was placed upside down using a spacer inside a cell culture dish. The cell culture dish was placed in a humidified incubator of 37 °C and 5% CO_2_. To prevent the evaporation of the DAC droplet, the bottom of the cell culture dish was loaded with 7 mL of PBS. Then, after the spheroid cultivation, the fibroblast-associated glioblastoma spheroids were embedded into collagen using the PAC with alignment stoppers to make a spheroid–hydrogel model.

### 2.6. Cell Viability Assay

For the live/dead assay, the glioblastoma multiforme U87-MG spheroids in the DAC were transferred to a new DAC with each of its well filled with 24 μL of PBS containing 20 μM Calcein-AM and 10 μM EthD-1 using the PAC. After incubating the spheroids for 30 min in a humidified incubator of 37 °C and 5% CO_2_, the spheroids were retrieved using the PAC and returned to a new DAC with each of its well filled with 24 μL of DMEM medium for further imaging. To stain the spheroids already embedded in a collagen drop for the cell viability assay, the cell medium used to immerse a CWC was entirely removed, and 5 mL of PBS was loaded into the cell culture dish to wash the cell models. Then, 1 mL of PBS containing 20 μM Calcein-AM and 10 μM EthD-1 was gently loaded onto the top surface of the CWC so that it penetrates through the type I collagen and stain the spheroids. The bright-field and fluorescent images were taken using a charge-coupled device (CCD) camera (DP72; Olympus, Tokyo, Japan) on a fluorescence microscope (IX51; Olympus).

### 2.7. Nuclei Staining of Spheroids

The Hoechst 33342 solution, which emits blue fluorescence when bound to double-stranded DNA, was used to stain the nucleic acid of the spheroids to distinguish the live cells. To make a working solution, 0.5 μL of Hoechst 33342 solution was mixed with 1 mL of cell medium. The spheroids cultured in the DAC were transferred using a PAC to a different DAC with each of its well filled with the Hoechst working solution. The transferred spheroids were incubated for 10 min in a humidified incubator of 37 °C and 5% CO_2_, then the spheroids were retrieved, washed with a PBS-loaded DAC, and finally reloaded into a different DAC with a fresh new media. The bright-field and fluorescent images were taken using a CCD camera on a fluorescence microscope.

### 2.8. Staining of the Fibroblast-Associated Glioblastoma Spheroid

The fibroblast-associated glioblastoma spheroids were imaged using a CCD camera on a fluorescence microscope by staining the cells at suspension level with CellTracker^TM^ before the formation of spheroids. The U87-MG cells were stained using CellTracker^TM^ Green CMFDA dye and the NHLF cells were stained using CellTracker^TM^ Red CMTPX dye. The working dye solutions of both dyes were made by dissolving the dye product with dimethyl sulfoxide to a final concentration of 10 mM, then mixing 1 μL of the mixture with 1 mL of serum-free DMEM medium. After removing the culture media of the suspended cells, the prepared working dye solutions were gently added, and the suspended cells with the working dye solutions were incubated for 30 min in a humidified incubator of 37 °C and 5% CO_2_. The working dye solutions were removed later, and the culture medium was added again. After that, the stained cells of both cell types were counted, diluted with an extra medium to meet the desired initial seeding concentration ratio, and loaded to the well of the DAC.

### 2.9. Invasion Assay

Since the human glioblastoma multiforme U87-MG cell line is very pro-invasive, the spheroids extend themselves to the surrounding microenvironment as they are cultured. In this research, the rat tail type I collagen, at which the spheroids were embedded, acted as the ECM surrounding the spheroids. All mono-cultured spheroids consisting of only U87-MG cells, and co-cultured spheroids consisting of both U87-MG and NHLF cells at different seeding concentrations were monitored using a CCD camera on a fluorescence microscope. In addition, the invasive behavior of the mono-cultured spheroids in different collagen concentrations was also monitored using a CCD camera on a fluorescence microscope (Supporting Figure S3). The bright-field images of the spheroids were captured for further image analysis of the invasion distance and area using ImageJ software (National Institutes of Health, Bethesda, MD, USA).

### 2.10. Spheroid Drug Treatment

The fibroblast-associated glioblastoma spheroid, which was cultured for 7 days in the DAC using the chip-based hanging drop method and embedded into a CWC with a PAC, was treated with 1 μM doxorubicin. The normal medium was replaced with a doxorubicin-containing medium, and the spheroids were monitored for up to 120 h. Another array of spheroids embedded in the collagen drops with a normal medium was also monitored up to 120 h for the control experiment.

### 2.11. Image Analysis

All the bright-field and fluorescence images were taken with a CCD camera on a fluorescence microscope were further analyzed using the ImageJ software. The scale of the images was set to convert the pixel unit to a micrometer. The diameter and area of the spheroid were measured by assuming the spheroid as a sphere. In the case of nucleic acid-stained spheroids, the color threshold was adjusted and the “analyze particles” tool was selected to automatically draw the outer circumference of the spheroid. The program was also used to measure the distance of how far the migratory glioblastoma cell invaded the ECM. The invasion distance is the length between the surface of the spheroid and the single cell that radiated off the most from the spheroid surface.

## 3. Results and Discussion

### 3.1. Design and Concept

**Figure 1:**
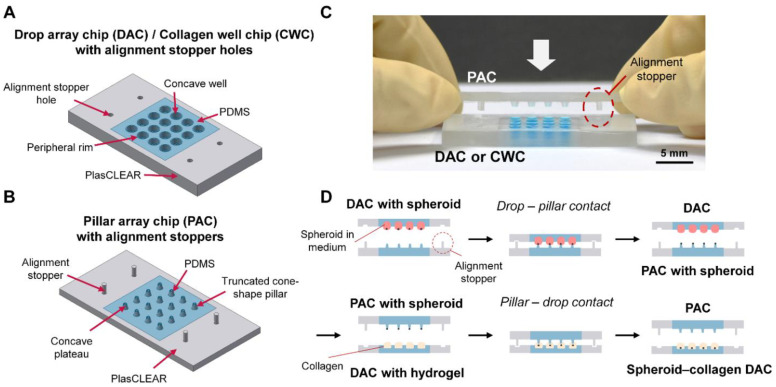
Schematic illustrations of the droplet-contact based spheroid transfer (DCST) devices and the working principle. Design of (**A**) the drop array chip (DAC) or collagen array chip (CWC) with alignment holes and (**B**) the pillar array chip (PAC) with alignment stoppers. (**C**) Image of the improved DCST method in action. (**D**) Working principle of the DAC with alignment stopper holes and the PAC with alignment stoppers using the improved DCST method.

A DAC with alignment stopper holes is used to culture spheroids using the chip-based hanging drop method (Figure 1A). The DAC consists of a PDMS-based concave well array assembled on the center of the plasCLEAR-based plate with alignment holes. A drop of cell medium or collagen is loaded into the concave well structure. Four alignment holes of the DAC were designed to fit the alignment stoppers of the PAC. The diameter of the well is set to be 2 mm and the wells are distanced away from each other by 4.5 mm. The wells also have a peripheral rim structure to prevent any liquid loaded into the well structure from spilling over. The dimension of the entire chip is 52 mm × 26 mm in length and width.

A PAC with alignment stoppers is used to hold and transfer spheroids using a pillar structure (Figure 1B). Similar to the DAC, the PAC also consists of a PDMS-based pillar array at the center of the plasCLEAR-based plate with alignment stoppers. The alignment stoppers, shaped like a long and thin cylinder, protrude from the surface to be fitted into the alignment holes of the DAC. The pillars have a truncated cone shape, with an upper diameter of 1 mm. The pillars are also distanced away from each other by 4.5 mm so that it aligns with the DAC. The top of the pillar has a concave plateau structure with air holes for a consistent amount of drop to be transferred onto the pillar and to prevent bubbles from being trapped within the pillar structure [24]. The dimension of the entire chip is 52 mm × 26 mm in length and width. The cylindrical alignment stoppers of the PAC are designed to perfectly fit into the alignment holes of the DAC (Figure 1C). At the same time, the array of the truncated cone-shaped pillars is positioned right over the array of the concave well structure, allowing uniform and simple transfer of spheroids. Owing to the lock-and-key structures of the two chips, no additional alignment process based on a microscope is required during the spheroid-transfer process, which enables a more convenient and less time-consuming experiment to be carried out.

### 3.2. Working Principle

Using the hanging drop method, the DAC with alignment stopper holes is used to culture spheroids. The spheroid-loaded DAC with alignment stopper holes is positioned above the PAC-alignment stoppers (Figure 1D). Using the alignment stopper, the DAC and PAC are contacted and released, transferring the array of spheroids onto the pillar structure. The spheroids are cultured for up to 7 days and the medium is exchanged every 48 h by inverting the PAC upside down and making PAC–DAC contact with another DAC containing newly loaded medium. With the help of the gravitational force, the spheroids positioned on top of the pillar structure are transferred into a fresh new medium drop. The underlying working principle of the improved DCST method using the DAC with alignment stopper holes and the PAC with alignment stoppers is similar to that of the previously reported method [24].

To embed the array of spheroids into the collagen drops for further investigation of the invasion characteristics, a similar procedure is conducted as exchanging the media. The PAC loaded with spheroids is flipped upside down and contacted with another DAC loaded with collagen drops. The spheroids are then embedded into the collagen drops, and the drops with spheroids are polymerized in an oven for 30 min. 

### 3.3. Comparison of the Uniformity Test in DCST and Manual Pipetting Methods

**Figure 2:**
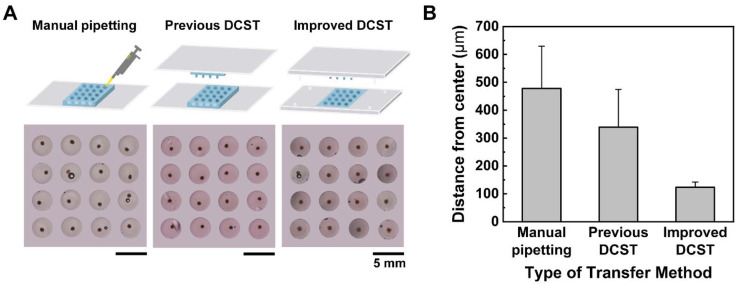
The comparison of the manual pipetting, the previous DCST method, and the improved DCST method. (**A**) Microscopic images of the array of spheroid–hydrogel models constructed using different spheroid transfer methods. (**B**) A bar graph of the average distance of the spheroid displaced from the center of the collagen drop.

The manual pipetting method, the previous DCST method, and the improved DCST method in this study were all compared to show the uniformity of the embedded spheroid models in collagen drops. A total of three arrays of the spheroid model were cultured using a hanging drop method, transferred into collagen drops using different methods, and the CWC was imaged from above. Both arrays of spheroids transferred using the manual pipetting method and the previous DCST method are inconsistently positioned, while the array of spheroids transferred using the improved DCST method are uniformly shaped (Figure 2A). Even at a glance, quantitative analysis was performed using the images by measuring the distance between the center of the collagen well and the center of the spheroid to show how far the spheroid is repositioned apart from the center of the well. The measured values were averaged and visualized into a bar chart (Figure 2B). Clearly, the spheroids transferred using the manual pipetting method are positioned most apart from the center of the well, and the previous DCST method follows. Most importantly, the standard deviation values of the manual pipetting method and the previous DCST method show that there is a large variation and inconsistency in the position of the spheroids among each array. The quantitative data indicate that the spheroids transferred using the improved DCST method were mostly centered and showed consistency among the models. Therefore, the improved DCST method allows a uniform construction of spheroid models and a more objective analysis of the invasive nature of the tumor spheroid models.

### 3.4. Comparison of the Spherical Shape of the Spheroid in the Improved DCST and Manual Pipetting Methods

**Figure 3:**
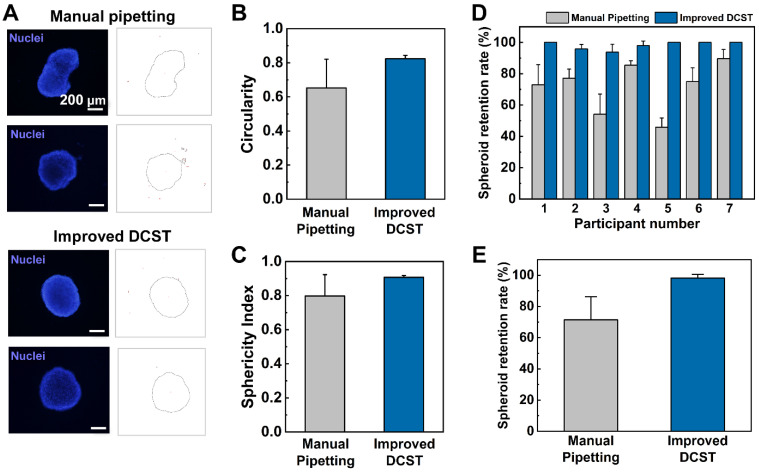
Characterization of the improved DCST method compared to the manual pipetting method. (**A**) Representative two images of the spheroids transferred by the manual pipetting method (left) and the improved DCST method (right). Blue: nuclei stained with Hoechst, black line: circumference of the spheroid. (**B**) A graph of circularity index of the improved DCST and manual pipetting methods. (**C**) A graph of the sphericity index of the improved DCST and manual pipetting methods. (**D**) A graph of the difference in spheroid retention rate between the improved DCST and manual pipetting methods of each participant. (**E**) The comparison of the average spheroid retention rate between the improved DCST and manual pipetting methods.

To prove the improved efficiency of the improved DCST device in terms of maintaining the spherical shape and viability of the spheroid drop, the spheroids were transferred using the improved DCST method and the manual pipetting method. The nuclei of the glioblastoma spheroids were then stained with a Hoechst 33342 solution, which has a blue fluorescence and imaged with a microscope to show any damage or breakage in the shape of the spheroid models. The fluorescent microscopic images and the modified circumference images of the spheroid models show that the improved DCST causes little to no damage to the spherical shape of the spheroid, while the manual pipetting method causes the spheroid to lose its original spherical shape and shatter (Figure 3A). For the spheroid models to be used for screening drugs or replacing animal testing, the spheroids must be maintained in whole and precise conditions. 

To further quantitatively compare the two transfer methods, the concepts of the circularity and sphericity index are introduced [25,26]. The circularity and the sphericity index equation are as follows:

(1) $$Circularity=\frac{4\pi \times Area}{{\left(Perimeter\right)}^{2}}$$

(2) $$Sphericity\;Index=\sqrt{Circularity}$$

Using the fluorescence image of the transferred spheroids, the area and perimeter were measured and inserted into each equation to calculate the circularity and sphericity index (Figure 3B,C). The circularity and the sphericity index have a range from 0 to 1, and the closer the value to 1, the more spherical the spheroid model. The values of circularity and sphericity index quantitatively show that the spheroid models transferred using the improved DCST method maintain their spherical shape and damages fewer number cells compared to the conventional manual pipetting method.

### 3.5. Comparison of the Retention Rate and Experiment Time

To compare the efficiency of spheroid transfer, the retention rates of using the improved DCST device and performing the manual pipetting method were calculated after performing an experiment with seven participants. Each participant carried out three trials of each method of transferring spheroids and the retention rate was calculated by counting the number of spheroids that were not lost or damaged and dividing the number of transferred spheroids by the initial number of spheroids. In each of the three trials, participants had to handle three arrays of 16 spheroids, for a total of 48 spheroids in one trial. The retention rate of each participant’s trial was averaged (Figure 3D), and then the results of all participants were averaged altogether and visualized as a bar graph (Figure 3E). The experiment clearly shows that the improved DCST method has a higher retention rate compared to manual pipetting, indicating that a smaller number of spheroids was damaged or lost. Compared to the conventional method, our device increases the spheroid retention rate by approximately 35%. Also, the manual pipetting method has a higher variation among different end-users, which may lead to higher inconsistency and lower reproducibility of experimental results. As the improved DCST method mainly uses gravitational forces to place the spheroids on top of the pillar, the method does not depend on the skill of the individual operator, while the manual pipetting method requires proficient operators in handling the spheroid transfer. Therefore, the improved DCST method is more user-friendly and efficient in transferring spheroids into a new medium or hydrogel compared to the conventional manual pipetting method.

Apart from the retention rate, the time required for the transfer of the spheroids was measured while the seven participants were performing the spheroid transfer experiment using the improved DCST and manual pipetting method. The manual pipetting method took 431.5 ± 114.3 s, while the improved DCST method took 60.5 ± 12.4 s, which means that the manual pipetting method took approximately seven times longer than the improved DCST method for the same spheroid transfer. The standard deviation values also suggest that the participants tend to be accustomed to the improved DCST method much faster and show similar performance in carrying out the experiment.

### 3.6. Growth and Analysis of Glioblastoma Spheroid

**Figure 4:**
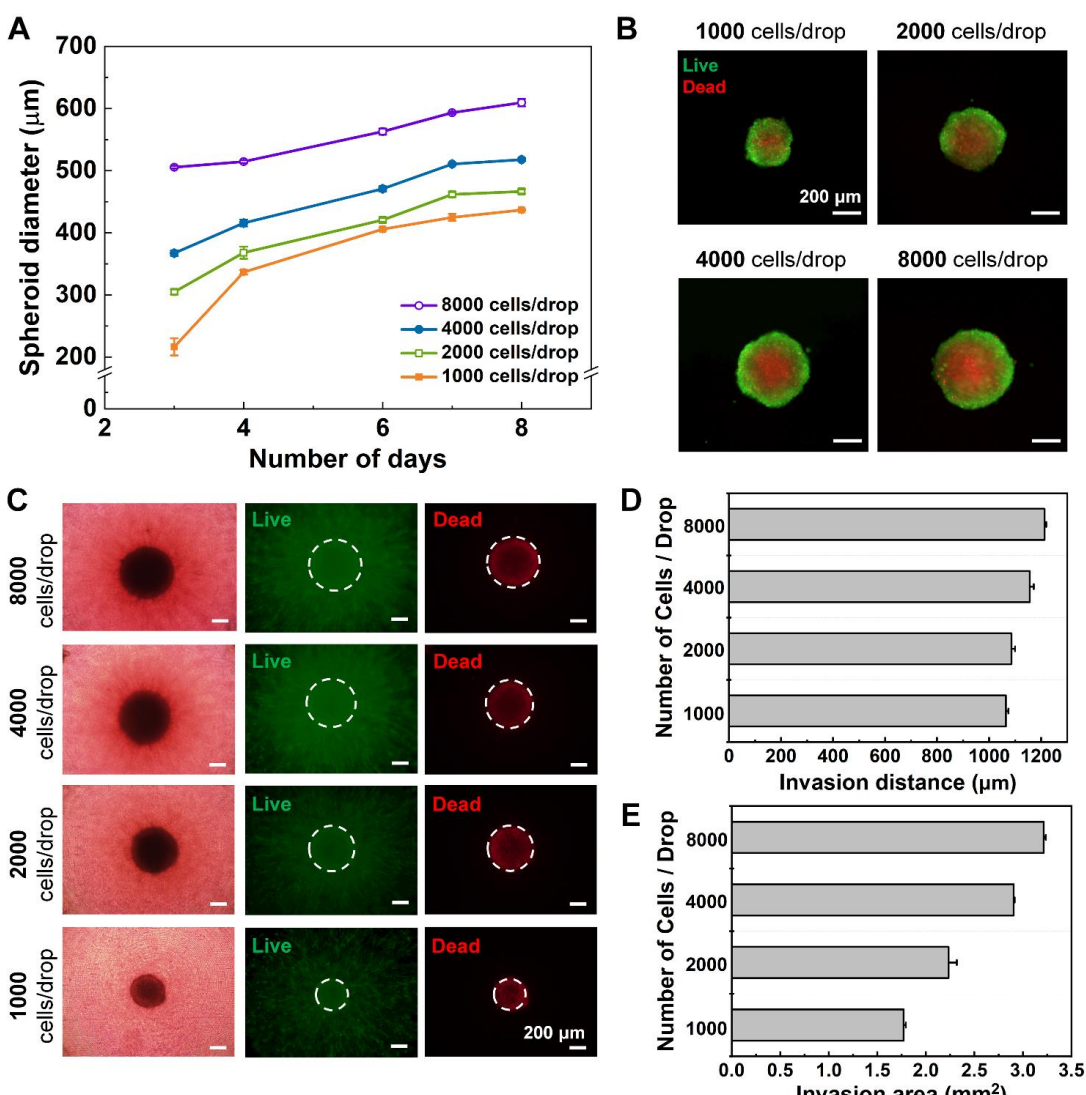
Growth and analysis of the invasive behavior of the glioblastoma multiforme spheroid using the improved DCST method. (**A**) A graph of the diameter of the spheroid of different initial seeding concentrations monitored for up to 8 days. (**B**) Fluorescent microscopic images of the spheroids of different seeding concentrations, stained with cell viability assay dyes to visualize the live (green) and dead (red) cells. (**C**) Microscopic images of the spheroid models embedded into collagen and cultured for 5 days. The spheroid models were stained with cell viability assay dyes to visualize the proliferated cells into the surrounding hydrogel. (**D**) A graph of the invasion distance of the spheroid–hydrogel models based on different initial seeding concentrations. (**E**) A graph of the invasion area of the spheroid–hydrogel models based on different initial seeding concentrations.

Using the improved DCST method, glioblastoma multiforme U87-MG spheroids were constructed in different sizes to find an optimal spheroid size suitable for an invasion assay. The four types of the initial seeding number of the glioblastoma spheroid are 1000, 2000, 4000, and 8000 cells per single medium drop. The U87-MG cell suspension was loaded to the DAC with alignment stopper holes with a different initial seeding number and cultured into spheroids and imaged for 8 days. The diameters were measured and show a consistent, increasing trend among the different seeding densities (Figure 4A). The spheroid diameter was directly proportional to the initial seeding concentration and culturing time. For the following experiments, spheroids seeded with a concentration of 4000 cells per drop were used, because they meet the criteria of having a diameter of approximately 500 μm or greater, which ensures the formation of a necrotic core inside the spheroid [27,28].

To confirm that necrotic cores were constructed within the spheroids, a live/dead assay using Calcein AM, and EthD-1 fluorescent dyes was performed, and the spheroids were imaged using a microscope (Figure 4B). Usually, in a spheroid, the cells located around the outer layer of the spheroids are mostly live as they can uptake the nutrients and oxygen from the surrounding environment, while the cells located near the core of the spheroids are mostly dead as the availability of oxygen and nutrients are low [10]. Such an observation was visible in all four seeding densities of spheroids, but more clearly visible in bigger spheroids with greater initial seeding concentration. The results show the capability of the DAC with alignment stopper holes to construct appropriate spheroid models of different seeding densities easily by simply manipulating the initial seeding concentration.

The spheroids grown in the DAC with alignment stopper holes for up to 7 days with different initial seeding concentrations were simultaneously transferred to the collagen drops using the PAC with alignment stoppers. The transferred spheroids settled in the middle of the well, and after collagen polymerization, the spheroid–collagen drops were immersed in a culture medium and placed in an incubator. After 5 days of culture inside the collagen drops, the spheroids stretched out into the neighboring collagen, which acts as an ECM because the invasive behavior was not clearly visible under the bright field, the spheroid-embedded collagen drops were stained with Calcein AM and EthD-1 (Figure 4C). The live cells that invaded the surrounding microenvironment were clearly visible under the fluorescent microscope. The images were quantitatively analyzed by measuring the distance of the farthest single cell from the surface of the spheroid and also the area of both the spheroid and the invaded cells to the neighboring collagen. The measured data were averaged and visualized into bar charts (Figure 4D,E). The number of initial cell seeding concentrations is directly proportional to the distance and area of the invasive nature of the glioblastoma spheroid. This can be explained by the direct relationship between the diameter of the spheroid and the initial cell concentration, as a greater initial seeding concentration means more active and live cells that can grow and also invade into the neighboring microenvironment. The results clearly show that the improved DCST method is better suited for the analysis of glioblastoma multiforme U87-MG spheroids, because the handling of the spheroids is much easier than the manual method, while the tumor spheroids show similar invasive behavior (data not shown).

### 3.7. Construction and Analysis of Fibroblast-Associated Glioblastoma Spheroid

**Figure 5:**
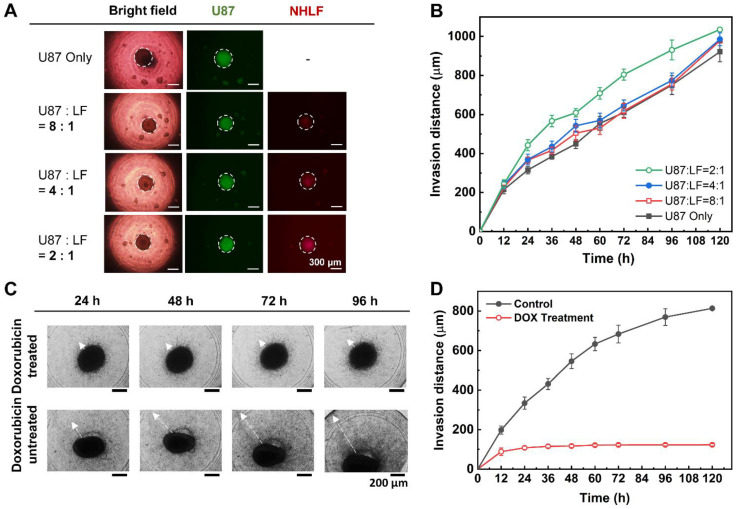
Growth and analysis of the invasive behavior of the fibroblast-associated spheroid using the improved DCST method. (**A**) Microscopic images of fibroblast-associated glioblastoma spheroids seeded at different concentrations, with glioblastoma cell stained with CMFDA (green) dye and fibroblast cell with CMTPX (red) dye. White dot circles indicate the outline of the spheroids. (**B**) A graph of the invasion distance of the fibroblast-associated glioblastoma spheroid with different initial seeding concentration ratios of the glioblastoma and fibroblast cell lines. (**C**) Microscopic images of the spheroid–hydrogel model treated with doxorubicin (DOX) and that of spheroid–hydrogel model not treated with the drug. (**D**) A graph of the comparison of the invasion distance of spheroid–hydrogel model treated with doxorubicin or not (control).

The U87-MG and NHLF cell lines were each stained with different colors (green and red) at the suspension level after trypsinization of the two-dimensionally cultured cells. Then, the cells were each counted and diluted with medium to meet the initial seeding concentration ratios of U87-MG:NHLF = 2:1, 4:1, and 8:1. Considering that fibroblast promotes the growth of tumor cells, the total seeding cell concentration was kept at 2000 cells per drop for each initial seeding concentration ratio of the co-cultured spheroids. After 7 days of culture in an incubator, the bright-field images under a microscope show spherically shaped spheroids (Figure 5A). The invasion distances of the spheroids of each initial seeding ratio embedded in collagen were measured and plotted along time (Figure 5B). It is known that cancer-associated fibroblast cells interact with the tumor cells to enhance the growth, proliferation, and invasion of the tumor [14]. The experimental results obtained show a similar tendency, as spheroids cultured with a greater amount of NHLF cells (U87-MG:NHLF = 2:1) have invaded into the neighboring tumor microenvironment the most among the four different seeding ratios. On the other side, spheroids cultured only with the glioblastoma cell for the control experiment tend to have a smaller invasion distance compared to other spheroids cultured with fibroblast cells. 

Furthermore, the fluorescent images of U87-MG cells in green and NHLF cells in red were taken to be analyzed. According to other studies, in the case of fibroblast-associated co-cultured spheroids, the fibroblast cells tend to grow at the center of the spheroid [29,30], and the tumor cells surround the fibroblast cells. A similar observation was seen in the fluorescent as the red NHLF cells were mostly positioned at the center of the spheroid, while the green U87-MG cells were located evenly in all areas of the spheroids (Figure 5A).

The fibroblast-associated glioblastoma spheroids that were cultured for 7 days in a DAC with alignment stopper holes using the hanging drop method were then transferred to a CWC using the PAC with alignment stoppers. Then, the spheroid-embedded collagen drops were treated with 1 μM doxorubicin, a well-known anti-cancer drug, and another array was not treated with any drug for a control experiment. The spheroids were observed for up to 120 h (5 days), regardless of whether they showed further growth or not (Figure 5C). It was observed with the bright-field images that the invasion of the spheroids was significantly reduced or even halted with the treatment of the drug, while the spheroids of the control group—the untreated fibroblast-associated glioblastoma spheroid—proliferated into the surrounding microenvironment. The invasion distance of both groups was measured and plotted onto a graph over time (Figure 5D). It is clear from the positively increasing trend line that the control group shows steady expansion into the surrounding collagen, but the flattening curve of the drug-treated group implies that the drug has penetrated the spheroids and stopped the invasive growth. Such verification and analysis on the effect of drug treatment on the invasive behavior of the fibroblast-associated tumor spheroids show the capability of the DAC–PAC alignment process as a simple and uniform method to construct a drug assessment model.

## 4. Conclusions

In this study, the uniform construction of an array of fibroblast-associated glioblastoma multiforme 3D cell culture models was demonstrated. The spheroids were grown in a medium droplet using the DAC with alignment stopper holes and transferred using the PAC with alignment stoppers by a DAC–PAC contacting process. This contacting process was used repetitively throughout the experiment for medium change, embedment into collagen as well as cell staining, and drug treatment. With the help of the alignment stoppers, the process of DAC–PAC contacting became more convenient by providing support and the constructed spheroid models were uniform. We were able to quantitatively demonstrate the enhanced performance of the improved DCST method in terms of the sphericity of the spheroid shape, higher retention rate, lower well-to-well variation. In addition, the experimental process of using the improved DCST method took less time than using the manual pipetting method. 

Using the improved DCST method, U87-MG spheroids were cultured and then analyzed both quantitatively and qualitatively with fluorescent microscope images to confirm proper spheroid growth and the existence of a necrotic core. Furthermore, fibroblast-associated tumor spheroid models were successfully cultured by mixing the glioblastoma multiforme U87-MG cell with the human lung fibroblast cell NHLF in different ratios. The fibroblast-associated tumor spheroid models were then embedded in collagen droplets and treated with a single concentration of doxorubicin to demonstrate the effects of drug treatment on spheroid invasion and to show the applicability of the spheroid models constructed using the improved DCST method as a drug assay platform. This study is expected to be applied in areas such as new drug development as a drug screening platform. Since the improved DCST method has wide applicability, it may also be used to conveniently construct and handle patient-derived tumor spheroids during the process of investigating a personalized cancer treatment.

## Data Availability

Not applicable.

## Supplementary Materials

The following are available online at https://www.mdpi.com/article/10.3390/bios11120506/s1. Figure S1. Schematic illustration of the fabrication process of the DAC with alignment stopper holes and the PAC with alignment stoppers, starting from printing the mods using a 3D printer to curing and peeling the devices from the molds. Figure S2. Comparison of a collagen well chip (CWC) with an original drop array chip (DAC). Figure S3. The effect of collagen concentration on the spheroid invasion.

## References

[1] Yamada K.M., Cukierman E. (2007). Modeling tissue morphogenesis and cancer in 3D. Cell.

[2] Swartz M.A., Iida N., Roberts E.W., Sangaletti S., Wong M.H., Yull F.E., Coussens L.M., DeClerck Y.A. (2012). Tumor microenvironment complexity: Emerging roles in cancer therapy. Cancer Res..

[3] Ko J., Ahn J., Kim S., Lee Y., Lee J., Park D., Jeon N.L. (2019). Tumor spheroid-on-a-chip: A standardized microfluidic culture platform for investigating tumor angiogenesis. Lab Chip.

[4] Huh D., Hamilton G.A., Ingber D.E. (2011). From 3D cell culture to organs-on-chips. Trends Cell Biol..

[5] Ong S.M., Zhang C., Toh Y.C., Kim S.H., Foo H.L., Tan C.H., van Noort D., Park S., Yu H. (2008). A gel-free 3D microfluidic cell culture system. Biomaterials.

[6] Loessner D., Stok K.S., Lutolf M.P., Hutmacher D.W., Clements J.A., Rizzi S.C. (2010). Bioengineered 3D platform to explore cell-ECM interactions and drug resistance of epithelial ovarian cancer cells. Biomaterials.

[7] Kim M.S., Yeon J.H., Park J.-K. (2007). A microfluidic platform for 3-dimensional cell culture and cell-based assays. Biomed. Microdevices.

[8] Fennema E., Rivron N., Rouwkema J., van Blitterswijk C., de Boer J. (2013). Spheroid culture as a tool for creating 3D complex tissues. Trends Biotechnol..

[9] Monteiro M.V., Gaspar V.M., Ferreira L.P., Mano J.F. (2020). Hydrogel 3D In vitro tumor models for screening cell aggregation mediated drug response. Biomater. Sci..

[10] Mehta G., Hsiao A.Y., Ingram M., Luker G.D., Takayama S. (2012). Opportunities and challenges for use of tumor spheroids as models to test drug delivery and efficacy. J. Control. Release.

[11] Lee J.M., Park D.Y., Yang L., Kim E.J., Ahrberg C.D., Lee K.B., Chung B.G. (2018). Generation of uniform-sized multicellular tumor spheroids using hydrogel microwells for advanced drug screening. Sci. Rep..

[12] Charoen K.M., Fallica B., Colson Y.L., Zaman M.H., Grinstaff M.W. (2014). Embedded multicellular spheroids as a biomimetic 3D cancer model for evaluating drug and drug-device combinations. Biomaterials.

[13] Truong H.H., de Sonneville J., Ghotra V.P.S., Xiong J., Price L., Hogendoorn P.C.W., Spaink H.H., van de Water B., Danen E.H.J. (2012). Automated microinjection of cell-polymer suspensions in 3D ECM scaffolds for high-throughput quantitative cancer invasion screens. Biomaterials.

[14] Jeong S.Y., Lee J.H., Shin Y., Chung S., Kuh H.J. (2016). Co-culture of tumor spheroids and fibroblasts in a collagen matrix-incorporated microfluidic chip mimics reciprocal activation in solid tumor microenvironment. PLoS ONE.

[15] Chaudhuri O., Koshy S.T., Da Cunha C.B., Shin J.W., Verbeke C.S., Allison K.H., Mooney D.J. (2014). Extracellular matrix stiffness and composition jointly regulate the induction of malignant phenotypes in mammary epithelium. Nat. Mater..

[16] Weaver V.M., Petersen O.W., Wang F., Larabell C.A., Briand P., Damsky C., Bissell M.J. (1997). Reversion of the malignant phenotype of human breast cells in three- dimensional culture and in vivo by integrin blocking antibodies. J. Cell Biol..

[17] Lang S.H., Sharrard R.M., Stark M., Villette J.M., Maitland N.J. (2001). Prostate epithelial cell lines form spheroids with evidence of glandular differentiation in three-dimensional Matrigel cultures. Br. J. Cancer.

[18] Li Y., Kumacheva E. (2018). Hydrogel microenvironments for cancer spheroid growth and drug screening. Sci. Adv..

[19] Nashimoto Y., Hayashi T., Kunita I., Nakamasu A., Torisawa Y.S., Nakayama M., Takigawa-Imamura H., Kotera H., Nishiyama K., Miura T. (2017). Integrating perfusable vascular networks with a three-dimensional tissue in a microfluidic device. Integr. Biol..

[20] Liao W., Wang J., Xu J., You F., Pan M., Xu X., Weng J., Han X., Li S., Li Y. (2019). High-throughput three-dimensional spheroid tumor model using a novel stamp-like tool. J. Tissue Eng..

[21] Han C., Takayama S., Park J. (2015). Formation and manipulation of cell spheroids using a density adjusted PEG/DEX aqueous two phase system. Sci. Rep..

[22] Kang E., Choi Y.Y., Jun Y., Chung B.G., Lee S.H. (2010). Development of a multi-layer microfluidic array chip to culture and replate uniform-sized embryoid bodies without manual cell retrieval. Lab Chip.

[23] Kim H., Cho C.H., Park J.-K. (2018). High-throughput culture and embedment of spheroid array using droplet contact-based spheroid transfer. Biomicrofluidics.

[24] Kim H., Roh H., Kim H., Park J.-K. (2021). Droplet contact-based spheroid transfer technique as a multi-step assay tool for spheroid arrays. Lab Chip.

[25] Kelm J.M., Timmins N.E., Brown C.J., Fussenegger M., Nielsen L.K. (2003). Method for generation of homogeneous multicellular tumor spheroids applicable to a wide variety of cell types. Biotechnol. Bioeng..

[26] Amaral R.L.F., Miranda M., Marcato P.D., Swiech K. (2017). Comparative analysis of 3D bladder tumor spheroids obtained by forced floating and hanging drop methods for drug screening. Front. Physiol..

[27] Zhang W., Li C., Baguley B.C., Zhou F., Zhou W., Shaw J.P., Wang Z., Wu Z., Liu J. (2016). Optimization of the formation of embedded multicellular spheroids of MCF-7 cells: How to reliably produce a biomimetic 3D model. Anal. Biochem..

[28] Xu X., Farach-Carson M.C., Jia X. (2014). Three-dimensional in vitro tumor models for cancer research and drug evaluation. Biotechnol. Adv..

[29] Lee S.W., Kwak H.S., Kang M.H., Park Y.Y., Jeong G.S. (2018). Fibroblast-associated tumour microenvironment induces vascular structure-networked tumouroid. Sci. Rep..

[30] Nashimoto Y., Okada R., Hanada S., Arima Y., Nishiyama K., Miura T., Yokokawa R. (2020). Vascularized cancer on a chip: The effect of perfusion on growth and drug delivery of tumor spheroid. Biomaterials.

