# Supplementary material for: Construction of a Fibroblast-Associated Tumor Spheroid Model Based on a Collagen Drop Array Chip
Source: Biosensors (Basel). 2021 Dec 9;11(12):506. doi: 10.3390/bios11120506 (PMC8699288; doi:10.3390/bios11120506)
Supplement: Supplementary file 1 [file biosensors-11-00506-s001.zip › biosensors-1475213-supplementary.pdf]

biosensors

Supplementary Materials

Construction of a Fibroblast-Associated Tumor Spheroid Model Based on a Collagen Drop Array Chip

Hyewon Roh [1], Hwisoo Kim [1] and Je-Kyun Park [1,2,*]

[1] Department of Bio and Brain Engineering, Korea Advanced Institute of Science and Technology (KAIST), 291 Daehak-ro, Yuseong-gu, Daejeon 34141, Korea; hyewonr@kaist.ac.kr (H.R.); hwiss@kaist.ac.kr (H.K.)
[2] KAIST Institute for Health Science and Technology, 291 Daehak-ro, Yuseong-gu, Daejeon 34141, Korea
* Correspondence: jekyun@kaist.ac.kr; Tel.: +82-42-350-4315

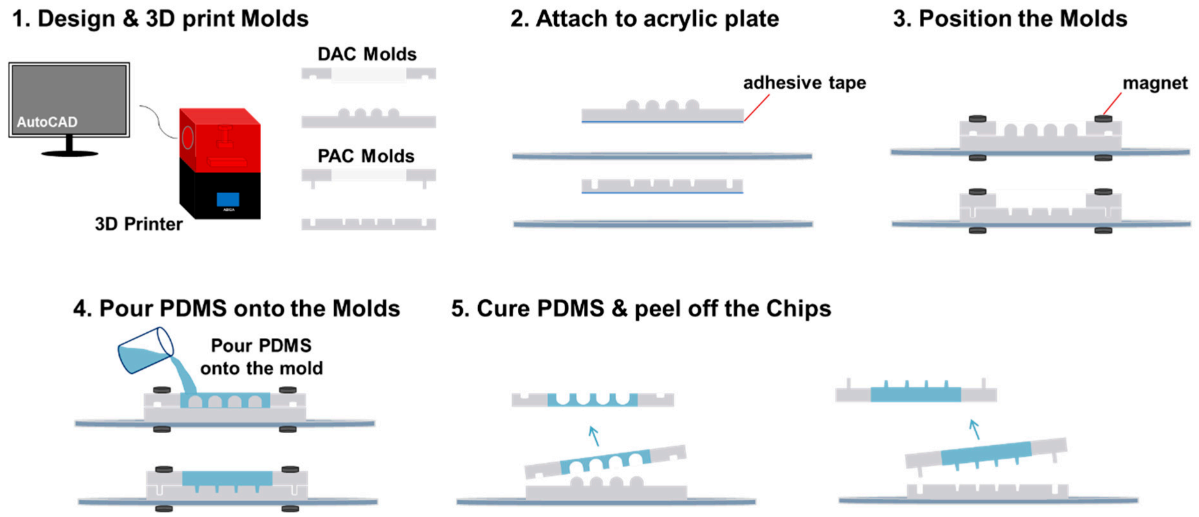

Figure S1. Schematic illustration of the fabrication process of the DAC with alignment stopper holes and the PAC with alignment stoppers, starting from printing the mods using a 3D printer to curing and peeling the devices from the molds.

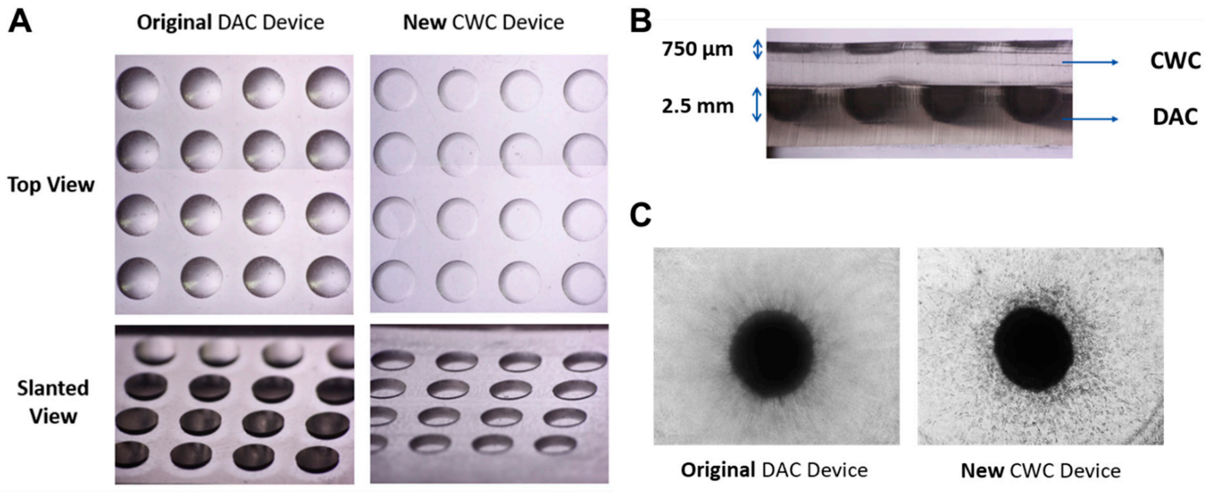

Figure S2. Comparison of a collagen well chip (CWC) with an original drop array chip (DAC). (**A**) Top and slanted views of the original DAC and the newly designed CWC. (**B**) Side view of the two devices. The height of the CWC is 750 µm, while the height of the DAC is 2.5 mm. (**C**) Invasion of the spheroid–hydrogel model in the original DAC and the new CWC.

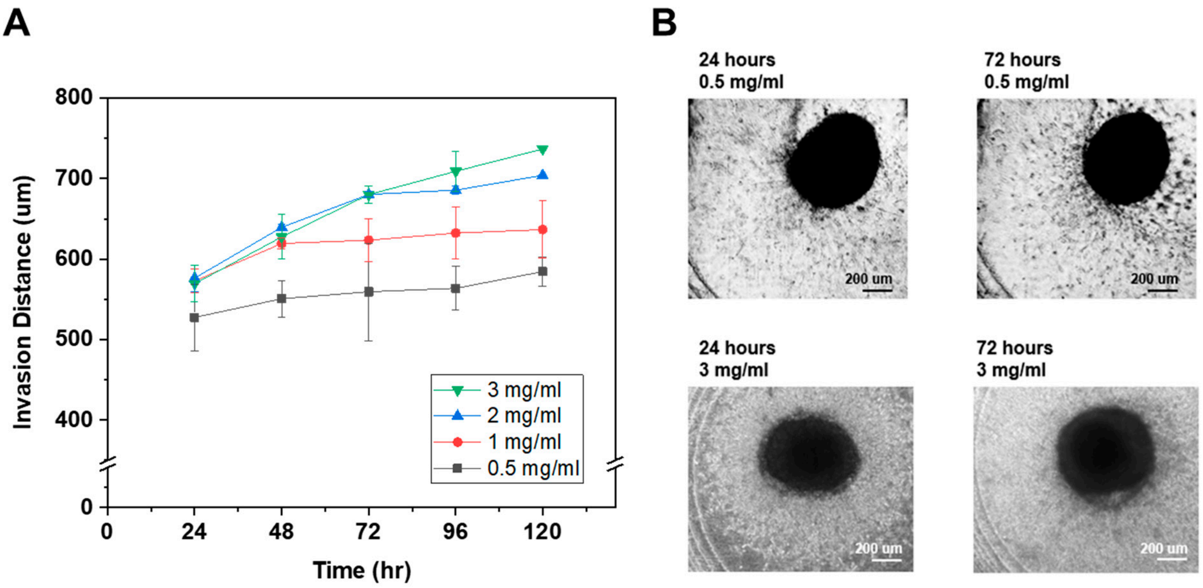

Figure S3. The effect of collagen concentration on the spheroid invasion. (**A**) The graph of invasion distance of the spheroids based on the concentration of the collagen. (**B**) The microscopic images showing the spheroid invasion at 24 h and 72 h under 0.5 mg/mL and 3 mg/mL.
